# Progress in Medicine

**Published:** 1900-07-07

**Authors:** 


### Progress in Medicine.

### RHEUMATISM AND GOUT.

(*Concluded from page* 205.)

Brockbank[12] notices the great importance of the so-called "growing pains," which may be the only symptoms of rheumatism in young people. Patients will often deny having had rheumatism or chorea, but will at once recollect pains under this name, which they imagined were natural to their age. However slight the arthritic symptoms may be, the heart may be affected. As D. B. Lees[13] remarks, in such cases there is generally dilatation, often a change in the first sound at the apex which may amount to a murmur, and a diffused impulse. In more serious cases the systolic apex murmur is followed by a double second sound.

A. Luff,[14] in discussing the diagnosis of rheumatoid arthritis, lays stress on its usually insidious commencement in one of the joints of the hands without redness or swelling at first, the subsequent symmetry, and bony outgrowths never seen in true rheumatism. Von Dungern and Schneider[15] record the discovery of certain small diplococci in the joints and gall-bladder of a patient who had suffered from rheumatoid arthritis. These, when cultivated and injected into the knees of a rabbit, produced the lesions found in the joints they came from. The reproduction of the disease in this manner completes the proof of the bacterial origin of the disease which Bannatyne and Wohlman brought forward a few years ago. A special form of the disease occurs in young children, and is marked by enlargement of the spleen and glands. As Taylor and McKine[16] remark, the bone ends and cartilages are rarely affected, and consequently there is no grating, but about the joints there is a smooth fusiform swelling and no osseous outgrowths. The sufferers are usually girls between their first and second dentition, and the disease is probably quite a distinct one. A. E. Garrod[17] proposes a classification of cases of rheumatoid arthritis under three groups: First appears the "nodular" variety, where Heberden's nodes and larger bony outgrowths are seen with but little swelling of the soft parts. Secondly, the fusiform variety, where the swellings affect both hands symmetrically, though there are no osseous outgrowths, and the terminal joints of the fingers usually escape. The spinal column is here attacked at an early period, and the temporo maxillary joints become stiff for a time. Thirdly, the crippling form of the disease, which commences in early adult life, tending to affect all joints and to stop all movements in those attacked, besides producing the most extraordinary deformities. W. Armstrong[18] suggests the following as a mode of classifying the various forms: (1) Acute; (2) subacute or chronic arthritis; (*a*) following or concurrent with true rheumatism; (*b*) following or concurrent with gout; (*c*) cases of utero-ovarian origin; (*d*) strumous or phthisical; (*e*) neurotic; (*f*) senile; (*g*) traumatic; (*h*) and toxic from gastro-intestinal fermentation or absorption. He adds that the prognosis of those cases which follow the gouty or rheumatic type is the more favourable, while that of the neurotic ones is the worst. Tonic treatment is desirable in all cases, and meat diet with little starch or sugar, while most patients find aid from the use of electric baths. G. Bannatyne,[19] on the other hand, thinks that (1) acute poly-articular arthritis, (2) chronic poly-articular, and (3) mono-articular arthritis are practically distinct diseases. Now the first occurs in young people, often in females, generally after some infective disorder. There are no bony outgrowths unless it goes on to the chronic form. This latter, when of a primary type, is most often seen in middle life, and is unaccompanied by fever, but the joints show numerous outgrowths and deposits. The mono-articular type occurs in elderly men, especially after an injury. The theory of a bacterial origin for the first of these forms is based on the frequency with which it follows an infective disease, and is followed by pleurisy and similar complaints, the presence of enlarged glands, and the appearance of nerve symptoms without any adequate nerve lesion existing. Lastly, we have the specific microbes cultivated from the affected joints, though Bannatyne confesses that he has not been able to reproduce the disease in animals. From the chronic forms of the disease it is difficult or impossible to isolate any organism at all, and the senile type probably depends on a simple degeneration of the tissues following an injury at a time of life when the nutrition of the parts is very inefficient.

With respect to treatment, he advises a plentiful diet, and such drugs as salol, guaiacol, and salicylate of quinine. He finds that the joints are relieved by painting with methyl salicylate, or guaiacol dissolved in oil, or in tincture of iodine. In the senile form baths, massage, and passive movements, with blisters and electricity, are useful.

The celebrated Aix-les-Bains treatment is described by H. Forestier,[20] who mentions that French authorities at the present time take nearly the same view as English writers on arthritis deformans. If the disease commences with an acute febrile attack, the baths should not be employed until that is over, or has continued some weeks. Then simple hot baths and local steaming is advised; but, as soon as the joints become less inflamed, gentle douche-massage is administered by two nurses for 10 minutes. This may be preceded on each occasion by a localised vapour bath or bath-tub, if thought advisable. Massage is given about 20 times in a month, but the course is varied according to the state of the patient, which in advanced cases may extend to two months, with very few applications of the douche-massage but more of the ordinary vapour baths.

**Pneumococcal Osteo arthritis** is recorded from time to time in France, but has not yet been seen in this country. Fernet and Lacapère[21] discuss a case where, as in all others of this disease, a rapid course with erosion of the bones and the formation of adhesions were noticed. The patient, a man aged 47, showed a swelling of the right wrist four days after the crisis of an attack of pneumonia. A fortnight later this was found to contain a clear fluid, in which the pneumococcus was present. Two months afterwards the radiogram showed that the bones were attacked, and the head of the radius was enlarged and eroded. Adhesions seemed to be formed between the neighbouring bones.

**Spondylitis.**—Besides the two recognised types of spinal ankylosis, Forestier[20] describes a pseudo neuralgic form. There is temporary rigidity of the spine, intercostal girdle pains, pains down the chief nerve trunks, and at the same time swelling of some peripheral joints. The disease is a curable one, and all these symptoms may disappear after treatment at Aix-les-Bains, or a similar spa. The writes describes five cases which were treated with success by him, and notices the curious posture and gait, the feet being often kept far apart, and the trunk held motionless. The neck, however, is not involved, as in the Bechterew type.

[12] Brit. Med Jour., April 28. [13] Brit. Med. Jour., Mar. 17. [14] Practitioner, May, p. 491. [15] Practitioner, May, p. 561. [16] Practitioner, May, p. 562. [17] Practitioner, May, p. 514. [18] Practioner, May, p. 512. [19] Practitioner, May, p. 530. [20] Practitioner, May, p. 544. [21] Lancet, June 16. [22] Med. Rec., April 14.

